# Ultrasound-guided microwave ablation for the treatment of abdominal wall scar endometriosis

**DOI:** 10.55730/1300-0144.6145

**Published:** 2025-12-20

**Authors:** Saffet ÖZTÜRK, Umut ASFUROĞLU, Esin KURTULUŞ ÖZTÜRK, Sadık Ahmet UYANIK, Gülcan KOCAOĞLU, İsmail Burak GÜLTEKİN, Hüseyin Levent KESKİN

**Affiliations:** 1 Division of Interventional Radiology, Department of Radiology, Ankara Etlik City Hospital, Ankara, Turkiye; 2 Department of Radiology, Ankara Etlik City Hospital, Ankara, Turkiye; 3 Department of Obstetrics and Gynecology, Ankara Etlik City Hospital, Ankara, Turkiye; 4 Department of Obstetrics and Gynecology, School of Medicine, Ufuk University, Ankara, Turkiye

**Keywords:** Abdominal wall endometriosis, cesarean scar, microwave ablation, minimally invasive procedure, ultrasound guidance

## Abstract

**Background/aim:**

To evaluate the efficacy of ultrasound (US)-guided microwave ablation (MWA) in treating abdominal wall endometriosis (AWE) lesions located at the cesarean scar and to assess the early clinical outcomes of this minimally invasive procedure.

**Materials and methods:**

A total of 18 patients diagnosed with AWE based on characteristic clinical and US findings were included in this retrospective study. Preprocedural visual analog scale (VAS) scores, ultrasonographic features, and lesion volumes were recorded. The procedure was performed under both general and local anesthesia to ensure patient comfort and procedural accuracy. Hydrodissection was performed before ablation to minimize the risk of thermal injury. Using US guidance, an MWA antenna was inserted into the AWE lesion, and ablation was performed with the moving-shot technique until the lesion became indistinct as microbubbles formed. Postprocedural VAS scores and lesion volumes were evaluated at 1, 3, and 6 months.

**Results:**

After MWA treatment, the median VAS score decreased from 8 (range: 8–10) to 0 (range: 0–1) at 1 month (p < 0.001). The mean volume of AWE lesions was 3.75 ± 2.42 cm^3^ before the procedure, 3.33 ± 2.1 cm^3^ at 1 month, 1.89 ± 1.18 cm^3^ at 3 months, and 0.75 ± 0.6 cm^3^ at 6 months, showing a significant reduction (p < 0.001). The longest axis and lesion volume progressively decreased during follow-up, with the most pronounced reduction observed at 6 months. Additionally, the volumetric reduction rate reached 79.6% at 6 months (p < 0.001). A minor infection occurred at the procedure site in one patient (5.6%) and was successfully treated with antibiotics.

**Conclusion:**

US-guided MWA significantly reduced cyclic pain (median VAS 8–10 to 0–1) and lesion volume at 6 months, with only minor complications observed. Larger, multicenter studies with extended follow-up are warranted to confirm the durability of these outcomes.

## 1. Introduction

Endometrial implants most commonly develop following cesarean delivery or hysterectomy. Less frequently, endometrial implants may occur along laparoscopic or thoracic tracts, within the amniocentesis needle tract, or at the site of a perineal incision scar. Implantation may occur within the subcutaneous tissue adjacent to the surgical scar or within the muscle layers, a condition referred to as scar endometriosis [1–3].

Abdominal wall endometriosis (AWE) is a rare form of extrapelvic endometriosis, most frequently located at the cesarean scar-site [4]. The reported incidence of AWE after cesarean delivery ranges from 0.03% to 0.8% [5]. Palpable nodular lesions at or around the cesarean scar and cyclic pain synchronized with the menstrual cycle are the most common symptoms, and this pain markedly impairs patients’ quality of life [6,7]. Therefore, accurate diagnosis and appropriate management are essential. Scar-site AWE is typically diagnosed based on characteristic clinical findings and imaging features, including ultrasonography (US) and magnetic resonance imaging (MRI). However, the gold standard for definitive diagnosis remains core biopsy [6]. Traditionally, treatment involves hormonal therapy or surgical excision. However, hormonal therapy has limitations, including the need for prolonged medication use and the frequent recurrence of symptoms after treatment discontinuation. Surgical excision remains the gold standard treatment, typically requiring a 5–10 mm surgical margin. Because of extensive tissue excision, mesh implantation or flap reconstruction may be required. Additionally, surgical procedures may be associated with complications such as wound eversion and fistula formation. Consequently, percutaneous minimally invasive treatments have gained increasing importance. Scar-site AWE can be managed using high-intensity focused ultrasound (HIFU), cryoablation, radiofrequency ablation (RFA), or microwave ablation (MWA) [8–14]. Only a few studies have reported the treatment of scar-site AWE using MWA [12,15,16]. Although evidence supporting image-guided ablation techniques is increasing, studies specifically evaluating MWA remain limited and are primarily small, single-center case series, highlighting the need for further research.

The primary objective of this study was to describe the MWA technique for treating cesarean-scar AWE lesions and to evaluate the early therapeutic efficacy of this minimally invasive ablation procedure.

## 2. Materials and methods

### 2.1. Study design and patient selection

Patients who underwent MWA treatment for AWE and were followed up between July 2024 and February 2025 were included in this study. Each case was jointly evaluated by interventional radiologists and gynecologic surgeons, and a multidisciplinary decision was made to proceed with MWA treatment. Before the procedure, all patients were informed about the MWA treatment, and potential complications were clearly explained. Written informed consent was obtained from all participants.

The study included menstruating women aged over 18 years who presented with one or more lesions and localized cyclic pain at or near a cesarean-scar site that affected their daily quality of life. Inclusion criteria also required characteristic clinical and ultrasonographic findings of abdominal wall endometriosis, such as a hypoechoic-to-heterogeneous solid lesion with ill-defined or blurred margins on ultrasound.

Exclusion criteria included premenopausal status, asymptomatic AWE lesions, a recent surgical history related to AWE, and contraindications to general anesthesia.

The diagnosis of AWE was established based on typical clinical findings and the detection of a hypoechoic lesion at or near the scar-site on ultrasound (US) [17]. Contrast-enhanced MRI and, if necessary, core biopsy were planned for patients with atypical clinical or ultrasonographic findings. However, all patients demonstrated typical clinical and ultrasonographic findings.

Technical success was defined as the complete hyperechoic transformation of AWE lesions during the procedure. Clinical success was defined as a postprocedural visual analogue scale (VAS) score between 0 and 1.

### 2.2. Preablation preparation

**Figure 1:**
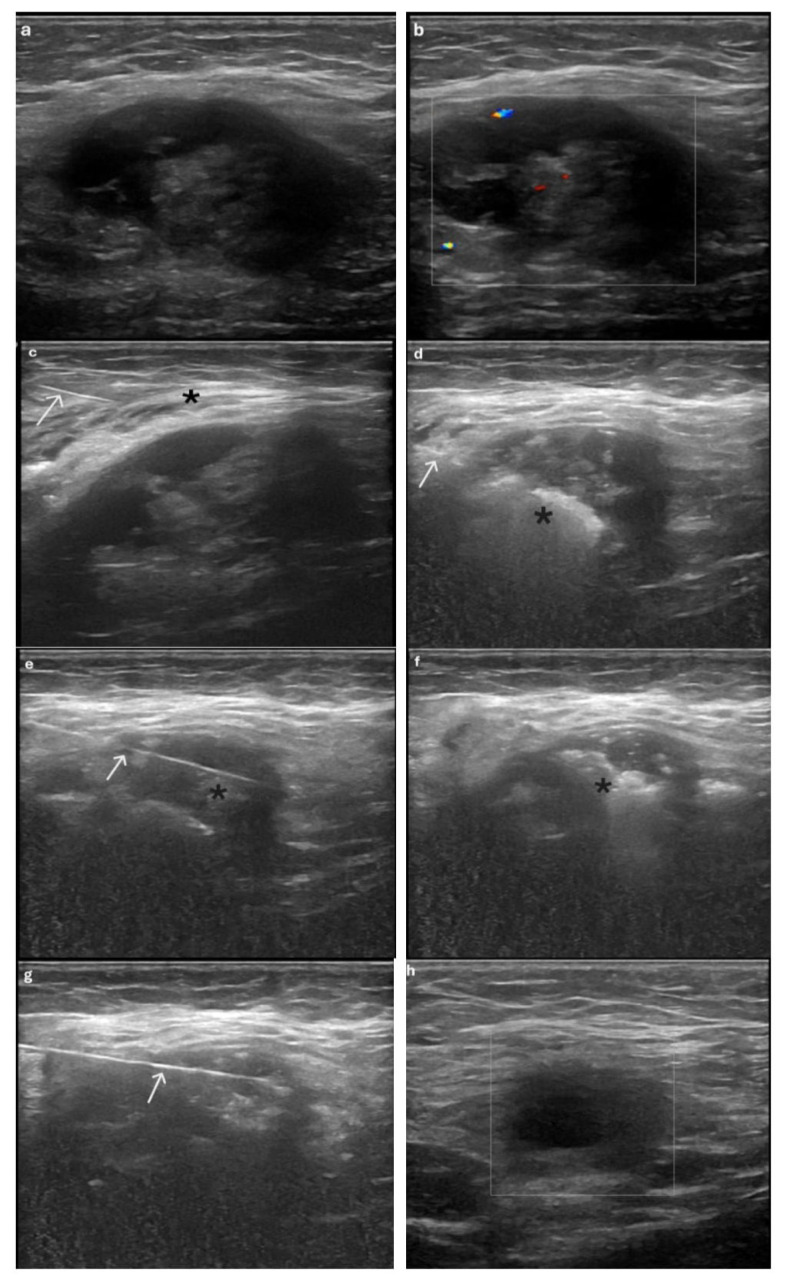
A 34-year-old female patient presenting with a palpable mass and cyclic abdominal wall pain on the right side of a cesarean section scar. (a) Ultrasound (US) image of the AWE lesion showing a heterogeneous mass with ill-defined, blurred outer margins. (b) Color Doppler US image demonstrating sparse vascular flow within the lesion. (c) Hydrodissection (asterisk) was performed anterior to the AWE lesion using 5% dextrose and a 21-gauge needle (arrow) under US guidance. (d–g) The microwave ablation antenna (arrow) was inserted into the lesion, and ablation was performed using the moving-shot technique. Gasification producing a hyperechoic cloud (asterisk) was observed during the procedure. (h) At the 6 month follow-up, the size and volume of the lesion were reduced, and color Doppler examination demonstrated absence of vascularity.

All patients were questioned about the use of antiplatelet and anticoagulant medications, and none reported using them. Before the procedure, data were collected on each patient’s VAS score, number of cesarean deliveries, date of the most recent cesarean, and any prior treatments received. AWE lesions were evaluated using US and color Doppler flow imaging (CDFI) (Figures 1a and 1b). The location, echogenicity, margins, size, and volume of AWE lesions were recorded under ultrasound guidance. Lesions confined to subcutaneous adipose tissue were classified as superficial, whereas those extending into the muscular layer were classified as deep. The longest axis (d_1_) and two perpendicular diameters (d_2_ and d_3_) were measured, and lesion volume was calculated using the ellipsoid formula: π × d_1_ × d_2_ × d_3_ ÷ 6.

### 2.3. Microwave ablation (MWA) procedure

All patients were positioned supine under general anesthesia, and the treatment area was prepared and sterilized. Local anesthesia was administered around the lesion using 10 mL of 2% prilocaine with a 21-gauge needle. General anesthesia was used in all procedures to maximize patient comfort, minimize movement during antenna placement, and ensure precise control during ablation. Under US guidance, the optimal puncture site and hydrodissection area for the AWE lesions were determined prior to the procedure (Figure 1c). Hydrodissection was performed around the AWE lesion using 5% dextrose injected into the subcutaneous fat layer. Posterior hydrodissection was not required, as a sufficient safety margin for ablation was already present. A total of 40–60 mL of 5% dextrose was used for hydrodissection. Preprocedural and postprocedural sonographic evaluations were performed using a 4–15 MHz ML6-15 linear transducer US system (LOGIQ P9; GE Healthcare, Chicago, USA). Subsequently, to prevent thermal injury to the skin and adjacent structures during ablation, 40–60 mL of 5% dextrose was infused around the lesion for hydrodissection. Under real-time US guidance, a 16-gauge microwave antenna with a 5 mm active tip was inserted into the AWE lesion using a KY-2000A microwave ablation system (Canyon Medical Inc., Nanjing, China). Ablation was performed at 40 W using the moving-shot technique. The ablation process was continuously monitored in real-time using US. Technical success was achieved when the entire lesion demonstrated a uniform hyperechoic appearance, after which the procedure was concluded (Figures 1e–1g).

The MWA procedure lasted for 60–192 s. To minimize potential postprocedural complications, patients were observed in the interventional radiology clinic for 4 h and then hospitalized overnight before discharge the following day.

### 2.4. Patient follow-up and outcome assessment

Patients were evaluated at 1, 3, and 6 months in the interventional radiology outpatient clinic, where VAS scores and AWE lesions were assessed using US (Figure 1h). As none of the patients reported pain suggestive of residual or recurrent disease, no additional imaging examinations were deemed necessary. The 1 month follow-up examination was scheduled after each patient’s first menstrual cycle following the procedure. Patients were advised to return to the interventional radiology clinic if they experienced any signs of infection, such as increased warmth, erythema, or swelling at the treatment site after the procedure. Additionally, minor and major complications were classified according to the Society of Interventional Radiology (SIR) standardized grading system [18].

### 2.5. Statistical analysis

Continuous variables were tested for normality using the Shapiro–Wilk test. Normally distributed variables were presented as mean ± SD and compared using the paired-samples t-test. Nonnormally distributed variables were reported as median (range). Repeated-measures analysis of variance (ANOVA) was used for post hoc evaluation of normally distributed data, whereas Friedman’s test was applied to nonnormally distributed data. Categorical variables were expressed as frequencies (percentages) and compared using the chi-square test. A p < 0.05 was considered statistically significant. All statistical analyses were performed using SPSS software, version 24.0 (IBM Corp., Armonk, NY, USA).

## 3. Results

A total of 18 female patients were included in the study, and 18 AWE lesions were treated with percutaneous MWA under US guidance. The mean age of the patients was 29.5 ± 4.17 years (range: 25–39 years), and all had a history of cesarean delivery. All cesarean scars were transverse. Among the participants, nine patients (50%) had undergone two cesarean deliveries, and two (11.1%) had undergone three. The mean interval between cesarean delivery and the onset of scar endometriosis symptoms was 2.62 ± 1.26 years (range: 1–5 years). All patients experienced cyclic pain corresponding to their menstrual cycle. In 10 patients (55.6%), a palpable nodular lesion was detected at the site of pain. Before the procedure, the median VAS score was 8 (range: 8–10) in all patients (Table 1).

In all AWE lesions, sparse vascularity was observed on color Doppler flow imaging (CDFI). All AWE lesions were located at or adjacent to the cesarean-scar level. In two patients, the AWE lesion infiltrated the rectus abdominis muscle and was deeply seated. In four patients, both deep and superficial components were identified. The sonographic features of the AWE lesions prior to treatment are summarized in Tables 2 and 3.

Following MWA treatment, VAS scores decreased to between 0 and 1 as early as the 1st month. In all patients, cyclic pain was significantly reduced or completely resolved (p < 0.001).

Therefore, MWA treatment was considered clinically successful. The mean volume of AWE lesions before treatment was 3.75 ± 2.42 cm^3^, decreasing to 3.33 ± 2.1 cm^3^ at 1 month, 1.89 ± 1.18 cm^3^ at 3 months, and 0.75 ± 0.6 cm^3^ at 6 months, demonstrating a significant reduction (p < 0.001).

**Figure 2:**
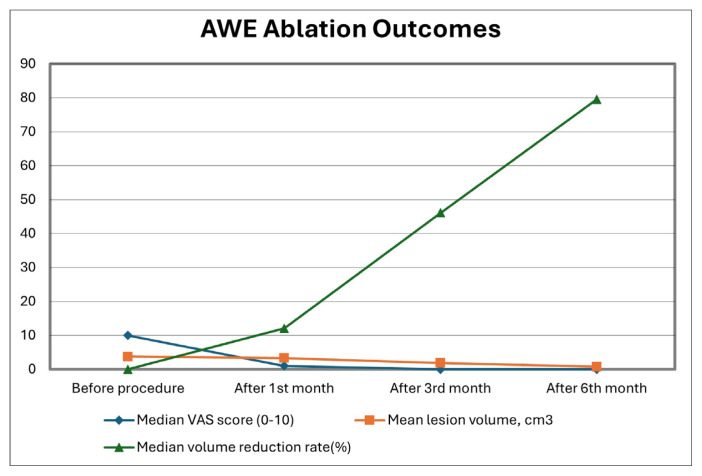
Clinical and volumetric outcomes of MWA in patients with abdominal wall endometriosis (AWE).

Both the longest axis and lesion volume decreased progressively during follow-up, with the greatest reduction observed at 6 months. Furthermore, the volumetric reduction rate reached its peak at 79.6% at 6 months (p < 0.001) (Table 4 and Figure 2).

Among the patients, one developed localized infection at the puncture site on the 4th postprocedural day, accompanied by mild fever, which was classified as a minor complication according to the SIR criteria. Symptoms resolved completely after a short course of antibiotic therapy. No major complications were observed in any patient. No recurrence or increase in VAS scores suggestive of disease recurrence was detected during follow-up in any patient.

## 4. Discussion

This study demonstrated that percutaneous MWA of AWE lesions under US guidance is an effective method for relieving cyclic abdominal pain and reducing lesion volume. From the first menstrual cycle after the procedure, cyclic pain decreased to a VAS score of 0–1, representing a significant reduction compared with preprocedural levels (p < 0.001). Lesion volume began to decrease from the 1 month follow-up, with the greatest reduction observed at 6 months, corresponding to a volumetric reduction rate of 79.6%.

Scar-site AWE typically presents with a palpable mass and pain at or around the surgical scar. The pain is usually cyclic, corresponding to the menstrual cycle. Because of its substantial impact on quality of life, accurate diagnosis and appropriate treatment are essential [6,7]. US and contrast-enhanced MRI assist in diagnosis, whereas histopathological confirmation remains the gold standard. US is a readily available and cost-effective imaging modality. In this study, the diagnosis of scar-site endometriosis was based on lesion localization at the cesarean-scar level, characteristic clinical findings (a palpable nodular lesion and severe cyclic pain), and supportive US features. Biopsy was not required because atypical clinical or sonographic findings were absent.

Treatment options for AWE include hormonal therapy, surgical excision, or percutaneous minimally invasive techniques [6]. Hormonal therapy has limitations, including potential drug-related side effects and the recurrence of symptoms shortly after discontinuation of treatment [19]. Surgical excision remains the first-line treatment, but it may necessitate mesh implantation or flap reconstruction in cases of extensive resection. Additionally, complications such as fistula formation, wound eversion, or reimplantation due to rupture of endometriotic nodules may occur [11,14,20]. Consequently, percutaneous minimally invasive treatment modalities have gained increasing clinical importance. AWE lesions can be managed using percutaneous minimally invasive modalities such as HIFU, cryoablation, RFA, and MWA.

Several studies have reported reductions in both VAS scores and lesion volume following cryoablation treatment for AWE lesions [14,21,22]. In the study by Jouffrieau et al., at the 6 month follow-up, eight patients (27.6%) had residual symptoms, and four patients (13.8%) had confirmed residual lesions on MRI [21]. Similarly, Bochour et al. reported that at 6 months, seven patients (18%) had VAS scores greater than 2, and four patients (11%) had residual lesions confirmed by MRI. The median procedure duration in that study was 62.5 min (range: 40–143 min) [14]. In the present study, no pain symptoms suggestive of residual lesions were observed at the 6 month follow-up. Therefore, contrast-enhanced MRI was not deemed necessary. Moreover, the mean procedure time was 126.6 ± 42.3 s (range: 60–192 s), which was considerably shorter than that reported for cryoablation.

Zhao et al. compared surgical excision and HIFU for AWE lesions, reporting that HIFU achieved comparable success to surgery in 25 treated patients. They reported that, aside from a few minor complications, HIFU caused neither blood loss nor tissue damage, unlike surgery. However, recurrence occurred in two patients (8%) [9]. In the present study, MWA proved effective for the treatment of AWE lesions. No major complications were observed, and only one patient developed localized erythema associated with a minor infectious process.

Mahdavi et al. evaluated 10 patients with AWE treated using RFA and reported decreases in both pain and lesion volume, indicating successful treatment. However, in two patients, although the VAS score decreased, it was found to be between 2–3 at the 6 month follow-up [13]. Similarly, in our study, both cyclic pain and AWE lesion volume decreased significantly. Moreover, the VAS scores of all our patients decreased to 0–1 as early as the 1st month.

Only a few studies have investigated the treatment of scar-site AWE using MWA [12,15,16]. Liu et al. evaluated nine patients and reported that pain resolved from the 1st month, with a volume reduction rate of approximately 68.7% at 12 months [12]. In the present study, a greater volume reduction rate was achieved in a shorter period, reaching 79.6% at 6 months. Furthermore, our study included a larger patient cohort, and VAS scores similarly decreased to 0–1 as early as the 1st month.

Yang et al. evaluated 30 patients and reported that treatment of AWE lesions with MWA under US guidance combined with single-port laparoscopy achieved the greatest volume reduction at 12 months. Additionally, recurrence was observed in five patients (16.7%) [16]. Treatment of AWE lesions with MWA can be performed as a minimally invasive procedure under imaging guidance. However, the use of a laparoscopic port does not fully comply with the principles of percutaneous minimally invasive therapy. In the present study, thermal ablation was safely and effectively performed using US guidance only. Moreover, despite a 6 month follow-up period, no recurrence was observed.

Image-guided procedures such as HIFU, cryoablation, RFA, and MWA have been shown to reduce pain and lesion volume in patients with AWE, without major complications. Therefore, percutaneous minimally invasive procedures performed under imaging guidance represent a viable alternative to surgical intervention.

This study is limited by its retrospective, single-center design, relatively small sample size, absence of systematic histopathological confirmation, and lack of long-term imaging follow-up for recurrence assessment. Prospective multicenter studies with larger patient cohorts are warranted to validate these findings. In conclusion, US-guided MWA in patients with symptomatic scar-site AWE resulted in complete resolution or a significant reduction of pain symptoms. A marked reduction in lesion volume was also observed, with no major complications reported. Therefore, MWA appears to be an effective and safe treatment option for patients with symptomatic scar-site AWE. However, studies with larger patient cohorts and longer follow-up durations are needed to confirm these findings.

## Figures and Tables

**Table 1:** Demographic and clinical characteristics of patients with AWE.

**Number of patients, n**	18

**Age, years**	29.5 ± 4.17 (25–39)

**Body Mass Index (BMI), kg/m** **^2^**	23.87 ± 3.8
**Groups, n (%)**	1(5.6)
<18.5 (underweight)	10(55.6)
18.5–24.9 (normal weight)	5(27.8)
25–29.9 (overweight)	2(11.1)
≥30 (obese)	

**Cesarean section incision**	1.53 ± 0.66
Number of cesarean incisions, n (%)	
1	9 (50)
2	7 (38.9)
3	2 (11.1)
Type of cesarean incision, n (%)	
Transverse	18 (100)
Vertical	0
Time interval from the last cesarean delivery, years	2.62 ± 1.26

Clinical signs and symptoms, **n (%)**	
Cyclic pain	18 (100)
Nodularity on palpation	10 (55.6)
**Visual analog scale (VAS) score**	10 (8–10)

**Ablation procedure**	
Ablation power, W	40 W
Ablation time, s	120 (60–192)

**Adverse effects within 24 h postprocedure, n (%)**	
Infection in the treatment site	1 (5.6)

Data are expressed as mean ± SD, median (interquartile range), or n (%), unless otherwise specified.

**Table 2:** Sonographic features of AWE lesions before treatment.

Lesion longest diameter, mm, range	22.54 ± 4.88

Lesion volume, cm^3^, range	3.75 ± 2.42

Location, n (%)	
Left side of the scar	12 (66.7)
Right side of the scar	5 (27.8)
Middle of the scar	1 (5.6)

Lesion depth,mm	8 (4–34)
Superficial/subcutaneous	12 (66.7)
Deep/involving the muscle layer	2 (11.1)
Involving superficial and deep layers	4 (22.2)

Shape, n (%)	
Round/oval	13 (72.2)
Stellate/irregular	5 (27.8)

Echotexture, n (%)	
Heterogeneous	15 (83.3)
Homogeneous hypoechogenicity	3 (16.7)

Margins, n (%)	
Ill-defined, blurred	18 (100)

Vascularization at color Doppler, n (%)	
Sparse vascular flow	18 (100)

Data are expressed as mean ± SD, median (interquartile range), or n (%), unless otherwise specified.

**Table 3:** Ultrasound (US) features of AWE lesions prior to ablation.

Lesion	Location (scar)	Depth	Distance from lesion to the skin (mm)	Echotexture	Margins	Shape	Vascularization at CDFI	Size (mm)
1	Left	Superficial	5	Heterogeneous	Blurred	Round	Sparse	21 × 17 x\× 27
2	Left	Superficial	10	Heterogeneous	Blurred	Round	Sparse	15 x\× 14 × 17
3	Left	Superficial	8	Heterogeneous	Blurred	Round	Sparse	14 × 11 × 19
4	Left	Superficial+Deep	18	Heterogeneous	Blurred	Irregular	Sparse	25 × 21 × 28
5	Left	Superficial	7	Heterogeneous	Blurred	Round	Sparse	18 × 14 × 21
6	Left	Superficial	5	Heterogeneous	Blurred	Round	Sparse	26 × 21 × 29
7	Left	Superficial+Deep	17	Heterogeneous	Blurred	Irregular	Sparse	26 × 17 × 24
8	Right	Superficial	4	Homogeneously hypoechoic	Blurred	Round	Sparse	17 × 13 × 15
9	Left	Deep	34	Heterogeneous	Blurred	Irregular	Sparse	22 × 14 × 25
10	Right	Superficial	9	Heterogeneous	Blurred	Round	Sparse	24 × 18 × 27
11	Right	Superficial	7	Homogeneously hypoechoic	Blurred	Round	Sparse	16 × 10 × 18
12	Left	Superficial	13	Heterogeneous	Blurred	Round	Sparse	16 × 14 × 26
13	Middle	Superficial	8	Heterogeneous	Blurred	Round	Sparse	14 × 11 × 17
14	Left	Superficial	13	Heterogeneous	Blurred	Round	Sparse	19 × 11 × 15
15	Right	Superficial+Deep	23	Heterogeneous	Blurred	Irregular	Sparse	38 × 21 × 36
16	Right	Superficial+Deep	18	Heterogeneous	Blurred	Irregular	Sparse	48 × 15 × 27
17	Left	Superficial	11	Homogeneously hypoechoic	Blurred	Round	Sparse	17 × 9 × 14
18	Left	Deep	12	Heterogeneous	Blurred	Round	Sparse	32 × 17× 21

**Table 4:** Preprocedural and postprocedural evaluation of MWA for AWE.

	Before procedure	^1^After procedure 1st month	^2^After procedure 3rd month	^3^After procedure 6th month	p value*	*Post hoc*
VAS score	10 (8–10)	1 (0–1)	0 (0–1)	0 (0–1)	<**0.001**	2.3<1
Lesion longest diameter (mm)	22.54 ± 4.88	21.61 ± 4.85	18.15 ± 4	13.5 ± 3.34	<**0.001**	1<2<3
Lesion volume, cm^3^	3.75 ± 2.42	3.33 ± 2.1	1.89 ± 1.18	0.75 ± 0.6	<**0.001**	1<2<3
Volume reduction ratio (%)	-	12.06 (3.96 –28.88)	46.10 (34 –67.1)	79.6 (40.7–96.38)	<**0.001**	1<2<3

* p < 0.05

Data are expressed as mean ± SD, median (interquartile range), unless otherwise specified.
